# Rethinking calcium profiles around single channels: the exponential and periodic calcium nanodomains

**DOI:** 10.1038/s41598-019-53095-4

**Published:** 2019-11-20

**Authors:** Sergej L. Mironov

**Affiliations:** Institute of Neuro- and Sensory Physiology, Georg-August-University, Göttingen, 37073 Germany

**Keywords:** Ion transport, Cellular neuroscience

## Abstract

Many fundamental calcium-dependent physiological processes are triggered by high local calcium levels that are established around the sites of calcium entry into the cell (channels). They are dubbed as calcium nanodomains but their exact profiles are still elusive. The concept of calcium nanodomains stems from a linear model of calcium diffusion and is only valid when calcium increases are smaller than the concentration of cytoplasmic buffers. Recent data indicates that much higher calcium levels cause buffer saturation. Therefore, I sought explicit solutions of a nonlinear reaction-diffusion model and found a dichotomous solution. For small fluxes, the steady state calcium profile is quasi-exponential, and when calcium exceeds buffer concentration a spatial periodicity appears. Analytical results are supported by Monte-Carlo simulations. I also imaged 1D- and radial calcium distributions around single α-synuclein channels in cell-free conditions. Measured Ca profiles are consistent with theoretical predictions. I propose that the periodic calcium patterns may well arise under certain conditions and their specific functional role has to be established.

## Introduction

Local Ca signals are important for cell signaling and often enable short-range signals to operate diverse activity-dependent events, such as synaptic transmission, neural plasticity, secretion and excitation–contraction coupling. Analytical treatments of Ca distributions within cytoplasm utilize mostly two models - a rapid binding approximation (RBA) and irreversible Ca binding. RBA assumes that after Ca entry into the cytoplasm, it equilibrates with cytoplasmic buffers. Analytical solutions are obtained after the model’s linearization and provide simple tools to interpret the experimental data. RBA dates back to Crank^1^ and postulates equilibrium of Ca and cytoplasmic buffers. A concept with different modifications and corrections has been applied to Ca reaction-diffusion in many papers, to mention but a few^2–9^. Of note, RBA application to experiments made in the same neuronal type, gave values of the intrinsic model parameter *κ ≈ B*_*o*_*/K*_*d*_ that vary by almost two orders of magnitude as summarized in^10^. This may merely manifest the fact that Ca reaction-diffusion systems are overdetermined, making it difficult to establish a molecular identity of Ca buffers in each particular case. In many instances, the measurements of cytoplasmic Ca are not consistent with assumption of a single Ca buffer and an additional immobile buffer is introduced. Whilst it is biochemically plausible, because the cytoplasm contains anionic substances such as membrane-associated protein residues and lipids, the identity of fixed buffers is not firmly established^9^. RBA is often linearized, which is applicable for free [Ca]< *K*_*d*_/2 i. e. for [Ca] < 0.25 µM, when the dissociation constant *K*_*d*_ = 0.5 μM (a median for Ca-binding proteins). Measured cytoplasmic Ca levels are usually larger, which requires the solution of non-linear RBA. A simple analytical model has been proposed^11^, which works well up to 100 µM Ca. It was also specified for the case of one mobile and one immobile buffer. The off-rate constant for Ca unbinding from the buffer can be estimated as *k*_*off*_ = *K*_*d*_^.^*k*_*on*_ = 100 s^−1^. Respective time constant *τ*_*off*_ ≈ 10 ms sets the time-scale for RBA applications >30 ms. RBA is appropriate to describe Ca-mediated reactions in compartments larger than 1 μm. They are bound by a membrane containing numerous (tens or hundreds) Ca channels and the boundary condition is determined by the average Ca current flowing into the compartment.

RBA is not appropriate for considering the fate of Ca immediately after its exit from the single channel. This was first recognized by Neher^12^ and now represents a cornerstone of the concept of Ca nanodomains as reviewed in^13,14^. This concept is functionally well suited to selectively activate low-affinity calcium sensors that are often strategically positioned in the immediate vicinity of single calcium channels. Neher used a simple linear model and considered steady state calcium profiles around a single calcium channel generated by the radial diffusion of calcium into the ‘infinite’ cytoplasm containing one buffer. He simplified a reaction-diffusion (RD) problem by assuming irreversible calcium binding and buffer excess. The assumptions reduce a non-linear problem to the linear ordinary differential equation (ODE)

1 $$D{C}_{rr}-({k}_{on}{B}_{o})C={0}$$

that describes a steady state distribution around a single Ca channel. Here *C* = [Ca]^.^*r*, *C*_*rr*_ is the second derivative with respect to the radial coordinate *r*.

The time-and space constants for irreversible Ca binding are defined after writing Eq. (1) into a dimensionless form. For a calcium diffusion coefficient of *D* = 220 µm^2^/s; the total concentration of cytoplasmic buffer *B*_*o*_ ≈ 0.2 mM and the on-rate constant for calcium binding *k*_*on*_ ≈ 2·10^8^ M^−1^s^−1^, the characteristic constants are *r*_*o*_ = √*D/k*_*on*_*B*_*o*_ = 70 nm and *τ*_*o*_ = 1*/k*_*on*_*B*_*o*_ = 40 μs, respectively. They are considerably smaller than those appropriate for RBA applications indicated above.

The solution of Eq. (1) for stationary radial Ca around the channel is

2 $$C(r)=A\,exp(-r/{r}_{o})$$

where the pre-exponential factor *A* defines the calcium level at the channel exit

3 $$A=i/2\pi DFR$$

where *i* is the single channel current, *R* is lumen radius and *F* is the Faraday constant. Justification of steady state Eq. (1) follows from the fact that *τ*_*o*_ is much smaller than the typical open and closed times of the calcium channels (>1 ms). Therefore, steady state profiles should be established fast after channel opening and quickly disappear after closure (see Supplement D for details). Equation (1) also assumes irreversible Ca binding, that is valid for *τ*_*o*_ ≪ *τ*_*off*_ ≈ 10 ms (see above).

A problem of linear treatment appears when we consider how large calcium levels around the channel may be. A theoretical estimate of the ratio *C*_*o*_*/i* is 1.2 mM/pA^15^. The value is in the range of recent experimental estimates obtained under certain experimental conditions ~0.7 (Tay *et al*. 2012) and 1.0 mM/pA^16,17^. When we take a single calcium current *i* ≈ 1 pA, the calcium level at channel lumen should be around 1 mM that would clearly exceed buffer concentrations and violate a simple linear model. This consideration prompted me to analyze a non-linear problem of steady state Ca profiles.

## Results

### Analytical solutions

Explicit solutions were derived for steady state distribution of Ca around the channel for irreversible Ca binding, without any restrictions on the magnitude of calcium influx or buffer saturation. Detailed analysis is made below for the case of single Ca buffer and extended to the two species in Supplements E and G.

It is convenient to work in the 1D-case, because the radial problem is readily transformed by dividing the calcium concentrations by the distance from the channel lumen (Supplement A). In all derivations below, the concentrations were normalized to the total buffer concentration *B*_*o*_. The times and distances are presented as *t* = *t/τ*_*o*_ and *x* = *x/r*_*o*_ (see the definition of the characteristic scales *τ*_*o*_ and *r*_*o*_ in Introduction). The RD problem for calcium and a single buffer are then given by the two partial ordinary differential equations (PDE)

5 $$\begin{array}{rcl}{c}_{t} & = & {c}_{xx}-cf+\gamma b\\ {f}_{t} & = & d{f}_{xx}-cf+\gamma b\end{array}$$

where small letters represent the normalized concentrations of free calcium (*c*), free (*f*) and Ca-bound (*b*) buffer, respectively, and *d* = *D*_*B*_*/D*_*Ca*_ is the buffer diffusion coefficient relative to that of calcium. The last term in the right-hand side represents calcium dissociation from the buffer. Subtracting the two equations in (5) produces a single PDE

6 $${(c-f)}_{t}={(c-df)}_{xx}$$

I first consider the case *d* = 1, when *D*_*B*_ = *D*_*Ca*_ (the assumption is inessential and removed below after Eq. (12) and also treated in Supplement E). The integration gives

7 $$c-f=A\,erfc(x/{2}\sqrt{t})-{1}$$

where *erfc* is the complementary error function. The constant *A* is defined by the boundary condition *c*_*x*=0_ = *A* = *C*_*o*_*/B*_*o*_, i. e. the calcium level at the origin divided by the total buffer concentration. Equation (7) helps to recast the system (5) into a single PDE

8 $${c}_{t}={c}_{xx}\mbox{--}c[c+1\mbox{--}A\,erfc(x/2\sqrt{t})]+\gamma b$$

I further neglect very small *γ* = *k*_*off/*_*k*_*on*_*B*_*o*_ = 0.005. As shown in Supplement F, its inclusion does not change the results of analysis.

Equation (8) can be presented in a more compact form after scaling the variables as *s* = *c/*|1 − *A|*, $z=x\surd |1-A|$ and *τ* = *t* |1 − *A|*, which gives

9 $${s}_{\tau }={s}_{zz}-{s}^{2}\pm s\beta erfc(z/2\sqrt{\tau })$$

with *ß* = *A/*|*1* − *A|*. The factor (*1* − *A*) can be either positive or negative (*A* = *1*, when [Ca]_*o*_ = *B*_*o*_).

Taking its modulus, a (±) sign accordingly appears in (9) before the linear term *sßerfc*. A single PDE (9) thus presents the two cases when the calcium level at channel lumen is greater or smaller than the buffer concentration, respectively.

The PDE (9) is non-linear and the solutions are analyzed in Supplement B. In the steady state solution *c*_*t*_ = *s*_*t*_ = 0 and *erfc* = 1. The corresponding ODE

10 $${s}_{zz}-{s}^{2}\pm \beta s={0}$$

already delivers important insights. For solving it, we integrate once

$${s}_{z}=s\sqrt{({2}s/{3}\pm \beta )}$$

and the second integration gives the two solutions

11a $$z=[\log \,\frac{\sqrt{(2s/3+\beta )}-\sqrt{\beta }}{\sqrt{(2s/3+\beta )}-\sqrt{\beta }}-w]/\sqrt{\beta },\,\,{\rm{f}}{\rm{o}}{\rm{r}}\,A* * *  < 1$$

11b $$z=2[arctan(\sqrt{(2s/3\beta +1})-w]/\sqrt{\beta },\,\,{\rm{f}}{\rm{o}}{\rm{r}}\,A > 1$$

where *w* is the constant of integration defined by the boundary condition *c*_*x*=*0*_ = *A*. After inverting (11) explicit expressions of the normalized calcium concentration read as

12a $$s=\frac{{3}/{2}}{sin{h}^{2}[(z+w)/{2}]}\,\,for\,A < {1}$$

12b $$s=\frac{{3}/{2}}{si{n}^{2}[(z+w)/{2}]}\,for\,A > {1}$$

The result is readily extended to the case when the diffusion coefficients for Ca and buffer are not equal. Let *d* = *D*_*B*_*/D*_*Ca*_ < 1 (the buffer diffuses slower than Ca). Equation (7) in the steady state implies that (*c* − *df*) = *const* = *A* − 1 and *f* = (*c* − *A* + 1)*/d*. This again produces Eq. (9), whose solutions are given by (12), but a spatial scaling in now *z* = *x√*(|1 − *A*|/*d*). This means a modification of the characteristic spatial scale, which becomes *r*_*o*_ = √*D*_*B*_/*k*_*on*_*B*_*o*_. Because *d* < 1, this should widen calcium gradients. In other words, in comparison to the linear model, the width of calcium nanodomains is determined by the diffusion coefficient of the buffer, not calcium. The notion is particularly important for bulky Ca binding proteins. Calmodulin and calbindin have e. g. *D*_*B*_ = 0.03*D*_*Ca*_ and the theoretical spatial scale should be around six-fold larger.

The non-linear RD problem is made here tractable by considering only one buffer, however, the cytoplasm may contain several calcium binding species. I treated the problem of the two buffers with different mobilities analytically in Supplement E. Fig. S3 shows that the decaying Ca nanodomain widens and periodic solutions demonstrate increasing spacing between Ca peaks that also gain amplitude. All these effects are consistent with the considerations above.

The on-rate constant *k*_*on*_ for calcium binding is usually diffusion-limited and around 2 · 10^8^ M^−1^s^−1^. The most notable exceptions are ‘slow’ buffers such as EGTA and parvalbumin discussed in Supplement C. The slow binding is due to the fact that the Ca binding sites are normally occupied by H^+^ (EGTA) or Mg^2+^ (parvalbumin) and the ions have to leave the binding pocket before Ca can be bound. For other buffers that bind Ca directly, the measured on-rates can vary by an order of magnitude^18^. In the linearized approximation given by Eq. (1), the effects of multiple buffers with different *k*_*on*_ values is easy to consider. They are all naturally pooled into the reaction term, such as (*k*_*on*_*B*_*o*_), which is replaced by the sum of products ∑*k*_*on,i*_*B*_*o,i*_ over all buffers that simply gives another constant. For a non-linear model (5) the situation is more complicated, because a single equation for Ca similar to Eq. (10) cannot be obtained with the help of the conservation rule (7).

I treated the case of two buffers with different on-rates analytically in Supplement G. Fig. S4 shows that the slow buffer only slightly modifies the Ca profiles established in the presence of fast binding buffer. From this follows that a buffer with the fastest Ca binding and highest mobility gives the main contribution to the steady state Ca profiles. Additional buffers only shape Ca patterns and their contribution can be accounted for as described in Supplements E and G.

### Numerical solutions and Monte-Carlo simulations

**Figure 1:**
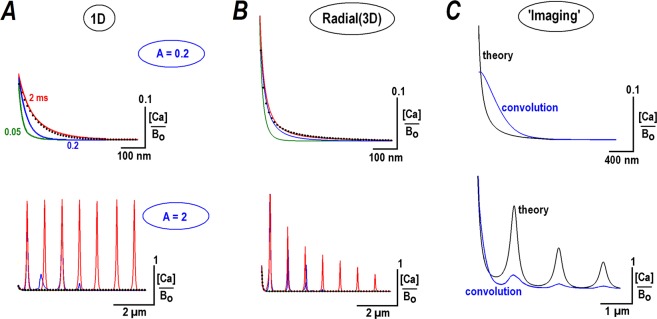
Steady state profiles for calcium diffusing from the point source (a single calcium channel). Calcium profiles were calculated for ‘calcium fluxes’ corresponding to the parameter *A* = 0.2 and 2, as defined in Eq. (7). The values were chosen to bracket the critical value [Ca]_*o*_/*B*_*o*_ = *A* = *1* that separates decaying and periodic solutions. Time-development of the profiles is presented by differently colored curves calculated at times of 0.05, 0.2, and 2 ms after switching on the calcium flux. The traces at 2 ms in 1D- (**A**) and radial (**B**) calcium distributions coincide with the steady state solutions of the non-linear RD equation (12). The black dots indicate the stationary exponential solutions predicted by linear treatment, Eq. (2). For *A* = 0.2, they coincide with theoretical predictions in the steady state and for *A* = 2, an exponential decay is only seen for very short times. (**C**) Simulation of how the radial calcium profiles would look in imaging. The theoretical curves (black) were convoluted with the Gaussian point spread function (HWHM = 0.4 µm). Note that the main peaks in the two panels are blurred and have widths around HWHM, but the secondary peaks for *A* = 2 are clearly resolved.

Next, I examined how the predicted steady state calcium profiles develop and solved Eq. (9) numerically (some analytical results are also presented in Supplement B). Figure 1A shows the convergence of the time-dependent 1D-solutions to the steady state profiles given by Eq. (12). For *A* = 0.2 the exponential profile is established within <1 ms. For *A* = 2 the initial pattern is decaying and then transforms into a spatially periodic waveform within <2 ms. Figure 1B shows similar calculations of the radial calcium profiles. They appear sharper, because, in comparison to a 1D-case, the concentrations are divided by the distance from the channel lumen (see Supplement A). As expected, the Ca gradients dissipate fast. Figure 2C in Supplement D demonstrates that initial peaks of Ca concentration become very small within 0.5 ms after switching off the influx of Ca. This substantiates the validity of assumptions of irreversible Ca binding and steady state analysis to examine nanodomains around single Ca channels.

**Figure 2:**
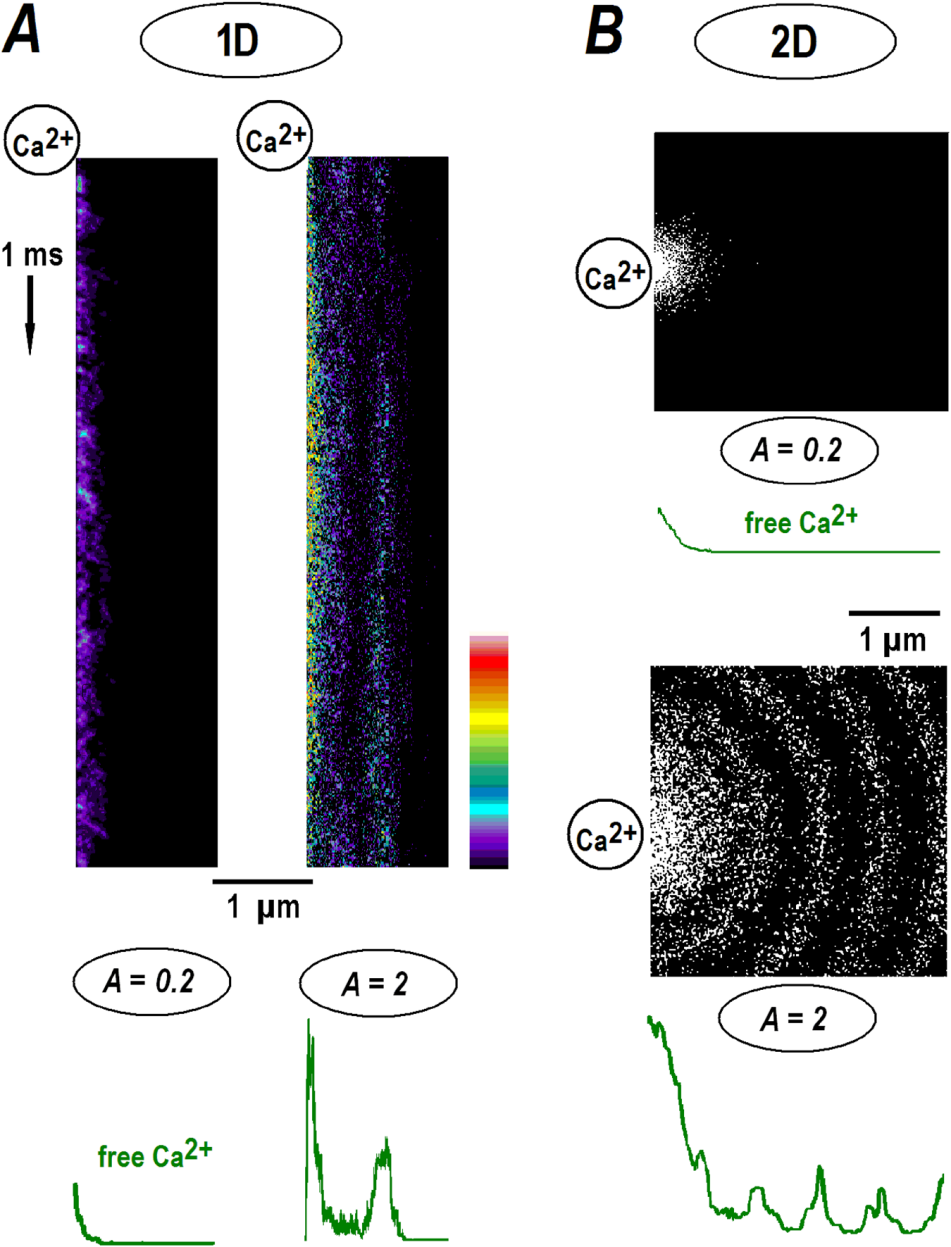
Stochastic modeling of calcium diffusion from the point source. Monte-Carlo simulations were performed as described in Methods. (**A**) The kymographs present the time-dependent changes in free calcium in the 1D-case for runs made for *A* = [Ca]_o_/*B*_*o*_ = 0.2 and 2, respectively (see also Fig. 1). The time direction is indicated by the arrow on the left. Calcium ions appeared randomly at the origin (the upper left corner), diffused, collided and reacted with free buffer molecules. The concentrations in the kymographs are pseudo-colored according to the calibration bar. The green profiles at the bottom show the mean 1D calcium distributions at the end of the runs. (**B**) Radial profiles for 2D-diffusion. The snapshots in the panels depict the positions of calcium ions (single dots) after 1 ms, close to the steady state (see also Fig. 1). Calcium levels at origin were set to *A* = 0.2 and 2 as indicated. Green curves below the panels show radial averages of calcium concentration in the end of the runs.

Calcium profiles in experiments are inevitably distorted due to finite imaging resolution. To simulate this, I convolved the radial profiles with the Gaussian point spread function *psf* = (*α/√π*)*exp*(*−α*^2^*x*^2^). The half-width-half-maximum (HWHM) was set to 0.4 µm, close to the experimental resolution of the imaging system (Methods). As expected, the ‘imaging’ decreases the amplitude and broadens calcium gradients (Fig. 1C). The decaying transients (*A* < 1) have a width around HWHM. The main peak in the periodic radial patterns (*A* > 1) is similarly blurred, but the secondary peaks are clearly discernable.

To further validate the analytical results, I simulated a stochastic diffusion of calcium in the presence of a buffer. The algorithm is described in Methods. Calcium ions appear randomly at the origin (*x* or *r* = 0) at a rate which sets the mean concentration at the channel lumen equal to a prescribed *A* value. Figure 2 presents sample simulation runs as kymographs, showing the instantaneous positions of free calcium ions (the locations of free and Ca-bound buffer molecules are omitted for clarity). In 1D-simulations the spatial profiles at the end of the run have a decaying exponential form (Fig. 2A) with a HWHM around 0.1 µm, close to the theoretical *r*_*o*_ value above. For *A* = 2, the main peak builds up fast and a secondary maximum takes more time to establish (Fig. 2A, the right panel). It is located at around 0.6 µm (=2*πr*_*o*_), a theoretically predicted position. In 2D-simulations the calcium distributions also showed either a decaying or periodic pattern, as determined by preset *A* (Fig. 2B).

### Imaging 1D- and radial calcium nanodomains

To test the theoretical predictions experimentally, I imaged calcium profiles around single α-synuclein (αS) channels in the excised patches. Such a cell-free experimental configuration is well suited for imaging calcium nanodomains, because the composition of the solutions bathing the membrane is well controlled. Fluo-4 was used, as the free probe has weak intrinsic fluorescence that increases >10-fold after calcium binding. Therefore, the regions without calcium binding marginally contribute to the measured fluorescence, which minimizes out-of-focus effects and improves the spatial resolution. The on-cell measurements, in contrast, are usually contaminated by bulk fluorescence that considerably mask weak local calcium increases.

**Figure 3:**
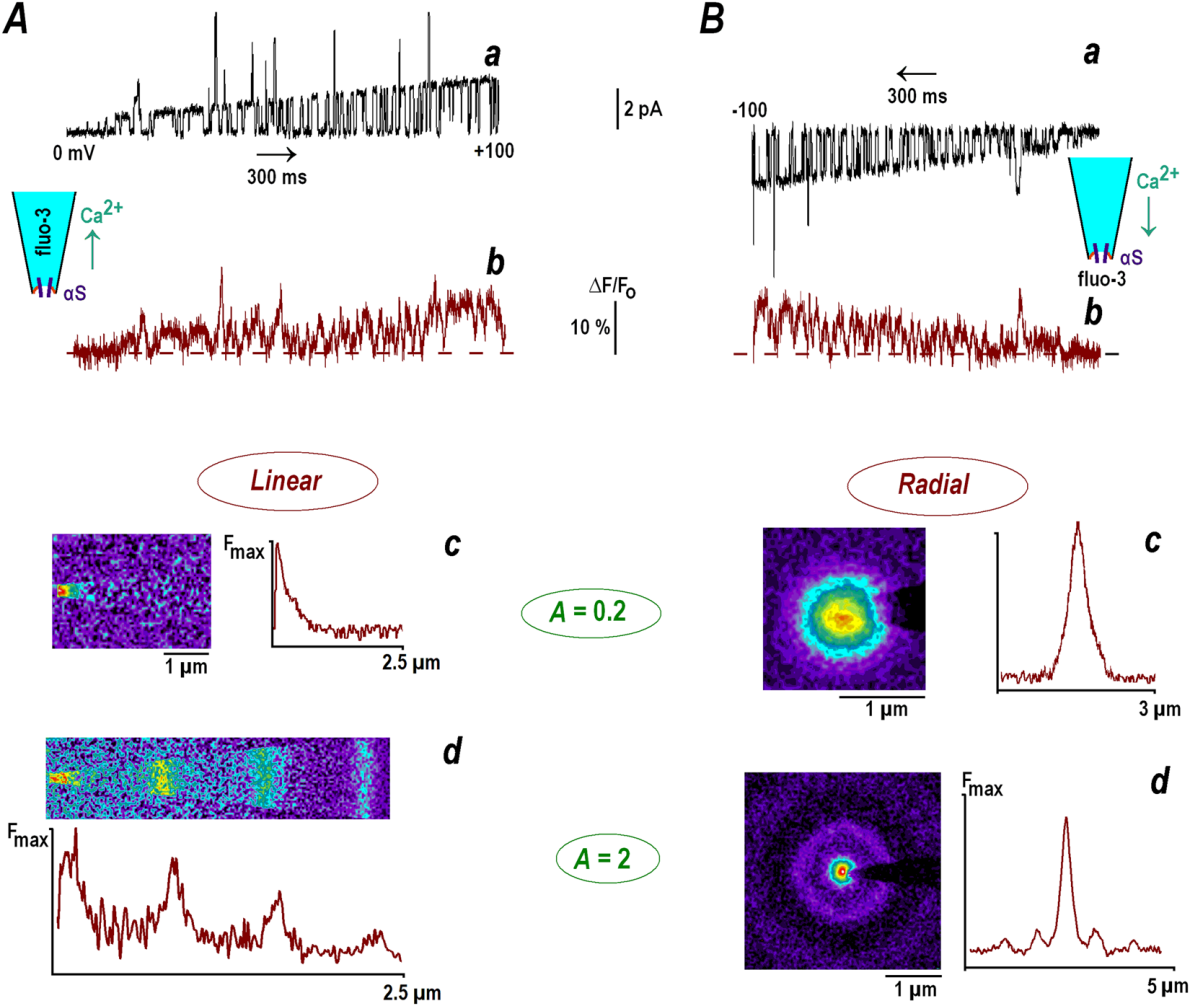
Calcium gradients around inside-out patches contained single α-synuclein channel. α-Synuclein (αS) channels were incorporated into the patches excised from cultured hippocampal neurons (see Methods and^18^). Successive panels (from the top downwards) present single channel calcium currents, calcium changes, average images at different patch potentials and their linear scans. (**A**) The uppermost trace shows channel opening during a 2 s-voltage ramp from 0 to +100 mV (**a**). The trace below depicts relative changes in mean fluo-4 fluorescence within a 2 µm-wide circle covering the patch (**b**). Pipette solution contained 150 µM fluo-4 in 154 mM NaCl and 88 mM CaCl_2_ was in the bath. Application of positive potential promoted calcium diffusion into the pipette (see inset at the top). The next two panels show average fluo-4 fluorescence (acquisition time, 3 s) at different patch potentials. The mean single channel current was 0.2 (**c**) and 2 pA (**d**). The values correspond to the theoretical parameter *A* = 0.2 and 2. Horizontal linescans (brown traces) present ‘one-dimensional’ stationary fluo-4 signals. The measured HWHM (half-width at half-maximum of the peak) was 0.43 µm at *A* = 0.2, close to the radial resolution of the experimental set-up (HWHM ≈ 0.4 µm, Methods). For *A* = 2 the calcium distribution showed periodic structure with equidistant peaks separated by 0.66 µm, in accord with a theoretical estimate of 2*πr*_*o*_ = 0.63 µm (see text). (**B**) Radial calcium profiles generated by the calcium currents through αS channels into the bath (see inset at the top right). The medium contained 150 µM fluo-4, and 88 mM CaCl_2_ was in the pipette. The top trace (**a**) shows channel activity during a 2 s-voltage ramp from 0 to −100 mV and the lower trace (**b**) presents mean changes in fluo-4 fluorescence around the pipette tip (2 µm-wide ROI). The fluorescence images in the panels were acquired at different patch potentials and the brown traces on the right present the linescans made across image diagonal. The half-width of the spot was 0.41 µm for *A* = 0.2 (**c**). The calcium distribution for *A* = 2 (**d**) also showed secondary peaks. The concentric shells have radius 0.68 and 1.41 µm, respectively; close to the theoretical estimates (see text).

Single αS channels provide a convenient tool to test theoretical predictions. They mainly show a single conductance level. Higher conductance states appear seldom and have very brief openings and do not contribute much to Ca changes (Fig. 3Aa,Ba). The opening and closing times for αS channels are 10 and 50 ms, respectively. This assures full development of a stationary pattern around the channel and dissipation after channel closure. Holding potential at preset levels allowed manipulating the parameter *A* within each experiment several times.

αS channels were incorporated into the membrane of hippocampal neurons as described previously^18^ and fluo-4 was imaged using TIRF excitation (Methods). The mean calcium levels around the inside-out patches followed channel opening (Fig. 3, *top panels*). Steady state calcium distributions were imaged at different holding potentials set to maintain prescribed values of the theoretical parameter *A*, the ratio between calcium levels at the channel lumen (Eq. (3)) and buffer concentration in the medium (150 µM fluo-4). Under the experimental conditions used, the values of *A* are numerically equal to the mean single channel current e. g. for *i* = 0.1 pA, *A* = 0.1 etc.

The one-dimensional profiles were generated by forcing calcium to diffuse from the bath into the pipette filled with fluo-4. Stationary calcium increases established fast and the type of pattern depended on preset *A* value. For *A* = 0.2 calcium was localized at the pipette tip and at *A* = 2 the periodic pattern was observed (Fig. 3A). Both Ca profiles are consistent with the theoretical predictions. The radial patterns were generated by forcing calcium to diffuse out of the pipette into the bath containing fluo-4. For *A* = 0.2 only a single spot around pipette tip was observed and for *A* = 2 it was complemented by a concentric shell (Fig. 3B).

For small single calcium currents (*A* = 0.2), the measured HWHM in the case of linear and radial diffusion were 0.42 ± 0.05 and 0.44 ± 0.06 µm, respectively (mean data from four patches in each case). A theoretical characteristic space constant is *r*_*o*_ = √*D*_*Fluo-*4_/*k*_*on*_[*Fluo*-*4*] = 100 nm (see above). Thus, true quasi-exponential calcium decay was apparently hidden within the optical spot, in line with ‘imaging’ simulations (Fig. 1C). For large calcium fluxes (*A* = 2), the ‘stationary’ patterns were periodic. In the 1D case the mean separation between equidistant peaks was 0.65 ± 0.03 µm (*n* = 4). For radial diffusion the distance between the main and secondary peak was 0.68 ± 0.04 µm (*n* = 4). Both values are in accord with the theoretical estimate of 2*πr*_*o*_ = 0.63 µm for the experimental conditions used.

## Discussion

The local calcium gradients around single channels (calcium nanodomains) were predicted by Neher^12^ who assumed irreversible Ca binding to a single cytoplasmic buffer. As argued in the Introduction, Ca dissociation can be important only on time-scales greater than 10 ms and should not contribute to Ca profiles that are established and extinguished fast (within 1 ms, Supplements D and F). Linearized approximation of a simple second order ODE (1) gives an exponential solution whose decay constant defines the nanodomain width^12^. The concept of such local Ca increases around single channels is currently widely accepted to interpret the calcium-triggered mechanisms of synaptic transmission^13,14,19^.

Unfortunately, the original derivation was based upon a linear reaction-diffusion model that is valid only when the calcium flux is small and the buffer is in excess. Recent experiments^16,17^ indicate that calcium levels under certain conditions near the channel lumen may reach 1 mM, which is greater than the concentration of putative cytoplasmic buffers (median, ~0.2 mM).

I also used the assumption about irreversible binding of calcium exiting from the channel by a cytoplasmic buffer, and treated a genuine non-linear problem, Eq. (10). The analytical solution gave either exponential or periodic profiles, whose appearance is determined by Ca flux through the channel. A dichotomous solution critically depends on the parameter *A* = [Ca]_*o*_/*B*_*o*_, the ratio between the calcium level at the channel lumen and buffer concentration e. g. for *i* = 1 pA and *B*_*o*_ = 0.2 mM, the value of *A* = 5 > 1. Figure 1A shows that when the calcium level at channel exit is less than the buffer concentration (case *A* < 1), the calcium concentration decays quasi-exponentially and resembles a classical solution of the linear model. For *A* > 1 the non-linear model predicts a maximum at the origin and several equidistant peaks.

This is a novel, unexpected and perhaps a counterintuitive finding. Indeed, it would be reasonable to assume that calcium distributions around the channel have the same waveform and are proportionally scaled according to the magnitude of calcium flux. However, a theoretical analysis does not support this mechanistic interpretation. When calcium levels at the channel lumen exceed the buffer concentration, a periodic pattern is established. The theoretical analysis is supported by the Monte-Carlo simulations (Fig. 2) and experiments (Fig. 3).

Mechanistically, the appearance of the two different patterns is understood from the structure of Eq. (10). Without the quadratic term the steady state equation is a simple ODE, *s*_*xx*_ ± *s* = 0. When the linear term is negative (*A* < 1), the solution is exponential and when it is positive (*A* > 1), the solution is trigonometric. The latter is to be discarded as unphysical, because the calcium concentration can never be negative. This artifact is yet counterbalanced after inclusion of the quadratic term that delivers a strictly positive solution, Eq. (12b).

The periodic solutions predicted do not violate the concept of calcium nanodomains but rather extend it. In addition to a main peak around the origin, secondary maxima appear at large calcium fluxes. They are better observe in 1D-systems, where all peaks have a constant height. This is the case when calcium diffuses through single αS-channels into a fine pipette (Fig. 3Ad). The radial periodic profiles are also observed for calcium diffusion into the bath (Fig. 3Bd). The location of secondary peaks in 1D- and 3D-cases are in good agreement with the theoretical predictions. Smearing of theoretical profiles using a custom point-spread function (PSF) broadens the peaks and decreases their amplitude (Fig. 1C). The spatial single calcium profiles measured in some cell types^20–23^ demonstrate main maximum and distinct shoulders that may hide secondary peaks predicted by a non-linear model.

What could be possible implications to the cell biology? In the living cells a radial diffusion of calcium from the channel has an exponential or periodic waveform multiplied by a hyperbolic factor (~1*/r*, Fig. 1). The effects dampen secondary peaks (Fig. 1B,C, the lower panel), but Ca increases may be large enough (Fig. 1) to trigger specific calcium-dependent events in the channel neighborhood. Some implications are worth to mention. The calcium sparks in muscle cells and neurons are generated by closely apposed IP_3_ receptors. Ca release from internal stores requires Ca for a full activation (Wang *et al*. 2004). The secondary calcium peaks are well suited to maintain a collective activity in the clusters of IP_3_ receptors. Another example is the asynchronous transmitter release. Its calcium dependence is still elusive^24^, but the synaptic vesicles (with respective calcium sensors) are only loosely coupled to calcium channels^25,26^. The estimates here show that such remote vesicles can be reached by the secondary calcium peaks even if the sensor is located 0.5 µm away. A loose coupling at such distances is also recently described for classical synaptic transmission in central CA3-CA1 synapses^27^.

I used single αS channels in the membrane of hippocampal neurons to test the model experimentally. The activity of channels was stable for >20 min, which allowed the current to be set by deliberately changing the holding potential several times to generate the single channel current to set a desired value of parameter *A*. The experiments with excised patches excluded possible effects of endogenous Ca buffers and complex geometry in the cytoplasm. Only one buffer (indicator fluo-4) was unilaterally present and bathing conditions were well controlled. The data obtained in the patch-clamp experiments support “the proof of theoretical principle” for appearance of decaying and periodic Ca patterns around the channel (Fig. 3).

The findings may be further extended to study single Ca transients in the living cells. Different types of calcium channels types have similar biophysical properties (gating and conductance). For N-type (CaV 2.2) and L-type (CaV 1.3) the Ca level at channel exit was estimated to be around 1 mM^16,17^. In studies in living cells the concerns should be made that the patches will be empty in many trials and in others several channels may be active; Ca channels are subject to run-down within several minutes, especially after excision. The work with the cell-attached patches can be also hindered by the fact that cytoplasm contains various buffers and may have complicated geometry in submembrane regions. Imaging of calcium nanodomains would also suffer from out-of-focus effects that lower imaging resolution. All this may complicate the experiments as well as data interpretation.

The analysis establishes a critical role of the parameter *A* = [Ca]_*o*_*/B*_*o*_. The experimental estimates of the ratio between the calcium level at the channel exit [Ca]_*o*_ and the single channel conductance (*i*) is ~1 mM/pA^16,17^. For median concentration of cytoplasmic buffers *B*_*o*_ = 0.2 mM, the critical value of *A* = 1 corresponds to *i* = 0.2 pA. In submembrane regions the concentrations may be different. For example, the negative surface charges near the channel would attract cations (calcium) and repel anions (buffer). The net result would be a higher [Ca]_o_ and a lower *B*_*o*,_ which would increase *A*. Thus, at the channel lumen, the critical value [Ca]_o_*/B*_*o*_ = 1 may be readily achieved at smaller calcium currents.

Another important result of the analytical treatment concerns the influence of the buffer diffusion coefficient on the width of calcium nanodomains as commented in paragraph after Eq. (12). The characteristic space scale *r*_*o*_ in the linear model estimated in the Introduction after Eq. (1), put *D*_*Ca*_ into front. Calcium indicator probes have diffusion coefficients close to that of calcium e. g. *D*_*Fluo-4*_/*D*_*Ca*_ = 0.5. Intrinsic cytoplasmic buffers are bulky proteins with much smaller diffusion coefficients e.g. *D*_*Calmodulin*_/*D*_*Ca*_ = 0.03. Therefore, it is highly likely that the synthetic probes underestimate the actual width of calcium gradients in the cytoplasm. In a native environment the calcium nanodomains should be more extended. It would also seem imperative to visualize Ca with genetically encoded calcium probes that have a diffusion coefficient close to that of intrinsic calcium binding proteins.

Ca binding proteins are reported to have different mobility and on-rate constants. If the buffer moves and binds faster than any other present, it gives a major contribution to Ca nanodomains. This may represent a starting point in the analysis and the effects of all other buffers can be treated analytically as delineated in Supplements E and G. Figures S3 and S4 show that addition of slowly moving Ca binding buffer does not markedly change the profiles. Ca binding proteins often demonstrate cooperative effects. Underlying conformational changes are important for calmodulin and some other buffers. The time scale of such reactions is >10 ms, much slower than Ca binding and channel opening. Such effects fit the time scale of RBA as discussed in the Introduction.

Periodic calcium profiles may not be as exotic as they may seem. A spatial periodicity is well-known in the reaction-diffusion field^28,29^. A seminal example is the Liesegang periodic patterns^30^ that can be produced within a test tube. There is a current theoretical and experimental interest in non-homogeneous RD patterns launched by advances and aims in nanotechnology. This may require revisiting our previous knowledge and some steps have already been undertaken^31,32^. Because the cells perform various functions at the nano- and microscales, we need to understand how specialized biochemical reactions proceed and integrate on complex nanoscales. Calcium-dependent RD systems set a framework for excitable media prone to oscillations and travelling waves, but may also form discrete patterns. For example, FLIM-based imaging recently revealed heterogeneous calcium landscapes in the cytoplasm^33^. These depend on age, preceding activity and forecast to unveil the novel fundamental aspects of brain cell physiology. This study indicates a possibility for the formation of extended patterns around single calcium channels that may play a role in this and other phenomena. In particular, it concerns the spatial organization of release machinery and presynaptic channels. The new data obtained in many recent studies^34–37^ presume that local Ca changes under certain conditions may have new, previously unrecognized physiological roles.

## Materials and Methods

### Ethical approval

All mouse experiments were approved and performed in accordance with guidelines and regulations by the local authority, the Lower Saxony State Office for Consumer Protection and Food Safety (Niedersächsisches Landesamt für Verbraucherschutz und Lebensmittelsicherheit), executed also at Georg-August-University, Göttingen. Briefly, all animals had free access from the shelter to water and food and every effort was made to minimize animal suffering and the number of animals used. For removal of tissues, animals were deeply anaesthetized with CO_2_ inhalation at fixed concentration and rapidly killed by cervical dislocation.

### Patch-clamp and imaging

Cultures of hippocampal neurons were prepared from 2- to 4-day-old mice as described previously^38^. Bath and pipette solutions contained 30 mM Tris buffer (pH 7.4), and 154 mM NaCl or 88 mM CaCl_2_. The solutions had an osmolality from 305 to 315 mosmol/l. Fluo-4 was from Invitrogen (Darmstadt, Germany) and the common chemicals were from Sigma (Deisenhofer, Germany).

Membrane currents were measured with an EPC-7 amplifier (ESF, Friedland, Germany). Patch-clamp pipettes were pulled to have a long shank (around 5 mm) and had 20 ± 3 MOhm resistance. Recorded traces were filtered at 3 kHz (−3 dB), and digitized at 10 kHz. The current and potential are presented according to conventions for intracellular recordings. For data obtained in the inside-out mode, the signs of current and potential were inverted.

Imaging was made with the 63x objective lens (N. A. 1.4) of an upright microscope (Axioscope 2, Zeiss). The fluorescence was excited by 488 nm light from a SLM Diodenlaser (Soliton, Gilching, Germany) and captured by a cooled CCD camera (BFI Optilas, Puchheim) operated under ANDOR software (500 × 500 pixels at 12 bit resolution). The laser beam was delivered from below at the angle appropriate to evoke TIRF. The spatial resolution of the experimental set-up was estimated by imaging fluorescent beads (40 nm diameter). Their half-width at half-maximum (HWHM) was 0.39 ± 0.03 µm (a mean from 12 objects).

Coverslips with hippocampal neurons were placed on the microscope stage. The inside-out patches were excised and, when they showed no activity of intrinsic ion channels, αS channels were incorporated into the membrane by applying external voltage pulses as described previously^18^. The experiments were done when only one channel was inserted into excised patches. This was assured by recording the channel activity for 10 s to seek for possible double openings. The open probability αS channels is around 0.2 and, for two channels in the patch the probability of simultaneous opening is around a square of this value (0.04). A double opening should be seen every 0.25 s, on average. When such event was observed the patches were discarded.

The pipette with excised patch was positioned nearly horizontally and carefully lowered down to the bottom. The approach was controlled by monitoring the resistance, similar to that used in scanning ion conductance microscopy^39^. The pipette stopped when the pipette resistance dropped by 1%, that indicated that the tip is <100 nm from the bottom, within a TIRF illumination layer.

The stationary calcium distributions were measured in the bath or within pipette, depending on the direction of calcium flux. It was set by the holding potential to obtain a prescribed stationary value. αS channels have three conductance states, but the upper two had only brief openings and did not contribute significantly to calcium changes. The imaging of 1D-calcium profiles was made with an isotonic calcium solution in the bath and 150 μM fluo-4 in the pipette contained NaCl and buffer (Fig. 3A). For the imaging the radial calcium profiles the ionic composition of the solutions was reversed and the indicator was in the bath (Fig. 3B). Calcium profiles were also measured using the outside-out patches and demonstrated similar patterns (*n* = 6, data not shown).

### Stochastic simulations

To test theoretical predictions I simulated calcium diffusion in the presence of buffer in one and two dimensions. The parameters of the model were *D*_*Ca*_ = 600 µm^2^/s, *D*_*Fluo-4*_ = 300 µm^2^/s and the on-rate-constant for calcium binding was *k*_*on*_ = 2 · 10^8^ M^−1^s^−1^. In 1D-simulations (Fig. 2A), a 1 μm-linear compartment was divided into 1000 cells each contained 9 buffer molecules. That corresponded to 0.15 mM buffer, the concentration used in the experiments. The time step was 10 ns. Calcium was injected at the single point (*x* = 0) at a rate set to establish the mean calcium at the origin at 0.03 or 0.3 mM. The values are numerically equal to the parameter *A*. Calcium ions, fluo-4 and bound calcium molecules jumped into randomly chosen direction during the simulation runs at a rate prescribed by respective diffusion time constants. When calcium and free buffer molecules appeared within the same cell, a Ca-buffer particle was formed with the probability determined by *k*_*on*_. Consideration of calcium unbinding did not modify the results of the simulation; in line with the theoretical estimates (see Introduction). Before starting the simulation, buffer molecules were set randomly, the system equilibrated for 1 ms and then calcium ‘influx’ was switched on. The time-dependent calcium patterns are plotted in Fig. 2 as kymographs. It shows also mean concentration profiles obtained as averages of 1000 frames at the end of the run. Two-dimensional radial diffusion was simulated in a square (Fig. 2B). Free buffer molecules at a mean concentration of 0.15 mM were first equilibrated for 1 ms and then calcium influx was switched on. Other parameters were the same as in the case of 1D-diffusion.

## Supplementary information

Supplementary information

## References

[1] Crank, J. The Mathematics of Diffusion. Oxford: Clarendon Press. (1956).

[2] Zhou Z, Neher E (1993). Mobile and immobile calcium buffers in bovine adrenal chromaffin cells. J. Physiol..

[3] Wagner J, Keizer J (1994). Effects of rapid buffers on Ca^2+^ diffusion and Ca oscillations. Biophys. J..

[4] Pape PC, Jong DS, Chandler WK (1995). Calcium release and its voltage dependence in frog cut muscle fibers equilibrated with 20 mM EGTA. J. Gen. Physiol..

[5] Smith GD, Dai L, Miura RM, Sherman A (1995). Asymptotic analysis of buffered calcium diffusion near a point source. SIAM J. Appl. Math..

[6] Falcke M (2004). Reading the patterns in living cells — the physics of Ca^2+^ signaling. Adv. Phys..

[7] Matveev V, Zucker RS, Sherman A (2004). Facilitation through buffer saturation: Constraints on endogenous buffering properties. Biophys. J.

[8] Gin E, Kirk V, Sneyd J (2006). A bifurcation analysis of calcium buffering. J. Theor. Biol..

[9] Schwaller B (2010). Cytosolic Ca^2+^ buffers. Cold Spring Harb Perspect Biol..

[10] Matthews EA, Dietrich D (2015). Buffer mobility and the regulation of neuronal calcium domains. Front. Cell Neurosci..

[11] Mironova LA, Mironov SL (2008). Approximate analytical time-dependent solutions to describe large-amplitude local calcium transients in the presence of buffers. Biophys. J..

[12] Neher E (1986). Concentration profiles of intracellular Ca^2+^ in the presence of diffusible chelator. Exp. Brain Res..

[13] Augustine GJ, Santamaria F, Tanaka K (2003). Local calcium signaling in neurons. Neuron.

[14] Eggermann E, Bucurenciu I, Goswami SP, Jonas P (2011). Nanodomain coupling between Ca^2+^ channels and sensors of exocytosis at fast mammalian synapses. Nat. Rev. Neurosci..

[15] Mironov SL (1990). Theoretical analysis of Ca^2+^ wave propagation along the surface of intracellular stores. J. Theor. Biol..

[16] Tay LH (2012). Nanodomain Ca^2+^ of Ca^2+^ channels detected by a tethered genetically encoded Ca^2+^ sensor. Nat. Commun..

[17] Tadross MR, Tsien RW, Yue DT (2013). Ca channel nanodomains boost local Ca amplitude. Proc. Natl. Acad. Sci. USA.

[18] Mironov SL (2015). α-Synuclein forms non-selective cation channels and stimulates ATP-sensitive potassium channels in hippocampal neurons. J. Physiol..

[19] Südhof TC (2017). Molecular neuroscience in the 21st century: A personal perspective. Neuron.

[20] Wang SQ (2004). microdomain calcium in muscle cells. Circ. Res..

[21] Demuro A, Parker I (2006). Imaging single-channel calcium microdomains. Cell Calcium..

[22] Cheng H, Lederer WJ (2008). Calcium sparks. Physiol Rev..

[23] Laezza F, Dingledine R (2011). Induction and expression rules of synaptic plasticity in hippocampal interneurons. Neuropharmacology.

[24] Kaeser PS, Regehr WG (2014). Molecular mechanisms for synchronous, asynchronous, and spontaneous neurotransmitter release. Annu. Rev. Physiol..

[25] Beaumont V, Llobet A, Lagnado L (2005). Expansion of calcium microdomains regulates fast exocytosis at a ribbon synapse. Proc. Natl. Acad. Sci. USA.

[26] Rozov A, Bolshakov AP, Valiullina-Rakhmatullina F (2019). The ever-growing puzzle of asynchronous release. Front. Cell Neurosci..

[27] Jensen TP (2019). Multiplex imaging relates quantal glutamate release to presynaptic Ca homeostasis at multiple synapses *in situ*. Nat Commun.

[28] Koch AJ, Meinhardt H (1994). Biological pattern-formation - from basic mechanisms to complex structures. Rev. Mod. Phys..

[29] Vanag VK, Epstein IR (2007). Localized patterns in reaction-diffusion systems. Chaos.

[30] Liesegang RE (1896). Uber einige Eigenschaften von Gallerten. Naturwiss. Wochenschr..

[31] Epstein IR, Xu B (2016). Reaction-diffusion processes at the nano- and microscales. Nature Nanotechnology.

[32] Halatek AJ, Frey E (2018). Rethinking pattern formation in reaction–diffusion systems. Nature Physics.

[33] Zheng K (2015). Time-resolved imaging reveals heterogeneous landscapes of nanomolar Ca^2+^ in neurons and astroglia. Neuron..

[34] Tang AH (2016). A trans-synaptic nanocolumn aligns neurotransmitter release to receptors. Nature.

[35] Timofeeva Y, Volynski KE (2015). Calmodulin as a major calcium buffer shaping vesicular release and short-term synaptic plasticity: facilitation through buffer dislocation. Front. Cell Neurosci..

[36] Scimemi A, Diamond JS (2012). The number and organization of Ca channels in the active zone shapes neurotransmitter release from Schaffer collateral synapses. J. Neurosci..

[37] Rozov A, Burnashev N (2016). Fast interaction between AMPA and NMDA receptors by intracellular calcium. Cell Calcium..

[38] Mironov SL (1995). Plasmalemmal and intracellular Ca pumps as main determinants of slow Ca buffering in rat hippocampal neurones. Neuropharmacology.

[39] Korchev YE, Negulyaev YA, Edwards CR, Vodyanoy I, Lab MJ (2000). Functional localization of single active ion channels on the surface of a living cell. Nat. Cell Biol..

