# Supplementary material for: Rethinking calcium profiles around single channels: the exponential and periodic calcium nanodomains
Source: Sci Rep. 2019 Nov 20;9:17196. doi: 10.1038/s41598-019-53095-4 (PMC6868209; doi:10.1038/s41598-019-53095-4)
Supplement: Supplementary file 1 — Supplementary information [file 41598_2019_53095_MOESM1_ESM.doc]

**Supplemental Material to**

**Rethinking calcium profiles around single channels: the exponential and periodic calcium nanodomains**

S. L. Mironov

Institute of Neuro- and Sensory Physiology, Georg-August-University, Göttingen 37073, Germany

*Short title:* Novel patterns of single calcium transients

*Corresponding author*: S. L. Mironov

Key words: Calcium, nanodomains, periodic patterns, analytical solutions; Monte-Carlo simulations; imaging; single calcium channels

E-mail: [smirono@gwdg.de](mailto:smirono@gwdg.de).

***A. Radial steady state calcium profiles***

The spread of calcium from single channels into the cytoplasm is described by radial diffusion from the point source into the infinite medium (Neher, 1986; Mironov, 1990; Stern, 1992). By analogy with Eq. (9), the normalized ODE for the steady state calcium profiles can be written as

*s_rr_ +* (*2/r*)*s_r_ = s^2^ ± βs* (A1)

where *r* is the radial coordinate. The normalized space and concentration variables are defined after Eq. (8) in the main text. This ODE differs from Eq. (10) for 1D-case by the term in the left-hand side that contains the first spatial derivative. It is eliminated after substituting *s = u/r* that gives

*u_rr_ = ±u + u^2^/r* (A2)

For the constant flux of calcium through the channel, the boundary condition is

*dC/dr = i/2πDFR^2^* (A3)

where *R* is the exit radius, *i* is single channel current, *D* is the diffusion coefficient and *F* is the Faraday constant. The aforementioned substitution *C = u/r* transforms (A3) into

*Rdu/dr – u = i/2πDF* (A4)

The first term on the left-hand side is small and can be neglected. This gives

*u_x=0_ = i/2πDFR = C_x=0_* (A5)

Remarkably, the condition (A5) is identical to the one used in the 1D-case, Eq. (3). (A2) would have the identical solutions, if the square term *u^2^/r* was not present. In order to estimate its influence, I presented the right-hand side as

*u_rr_ =* (±*u + u^2^*) *+ µu^2^*(*1/r - 1*) (A6)

and expanded *u = u + µU*. The equation in zero order (*µ^o^*) is identical to 1D-equation (10) whose solution is given by (12). I estimated the first-order correction (*µ^1^U*) from the inhomogeneous ODE

*U_rr_* - (±*1* + *2u*)*U* = *u^2^*(*1/r - 1*) (A7)

using the shooting method. Fig. S1 shows that for both decaying and periodic solutions the corrections due to the first order are very small. This is valid for all values of *A* = *C_o_/B_o_* used. 1D-solutions thus appear sufficiently accurate to obtain radial calcium concentrations after simple division of [Ca] by the distance from origin, *r.*

Stern, M. D. 1992. Buffering of calcium in the vicinity of a channel pore. Cell Calcium 13, 183-192.


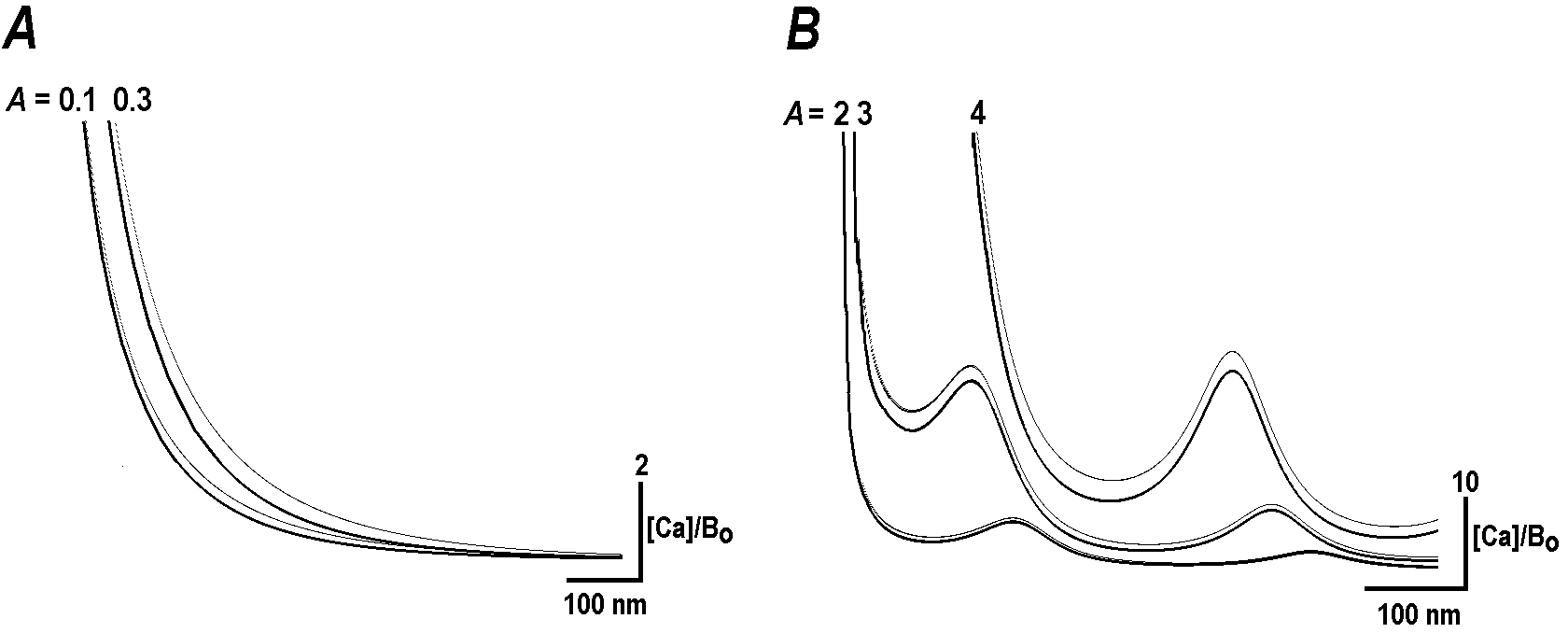


**Fig. S1. Radial calcium profiles.**

Thick curves represent the leading (zero order) term in Eq. (A2). Dotted curves show modifications after inclusion of the first order correction obtained through Eq. (A7). The calculations were made for cases when the calcium level at origin is smaller (***A***) and bigger (***B***) than the concentration of buffer (*B_o_*), respectively. The values *A* = *C_x=0_/B_o_* are indicated next to the respective curves.

***B. Time-dependent solutions***

The asymptotic analysis of PDE (9) in the RD field can be done by setting *s_t_* = *0* and leaving the time-dependent term *erfc*(*x/2√t*). This produces the non-linear time-dependent Schrödinger equation

*s_zz_ - s^2^ ± s ßerfc*(*y*) = *0* (B1)

Here *y* = *x/2√t* denotes a classical Boltzmann similarity variable that naturally appears in various diffusion problems (Polyanin & Zajtsev, 2012). In the WKB approximation (Holmes, 1995) the solution is

*Aexp*[-∫*√±erfc*(*y*)*dz*] + *Bexp*[∫*√±erfc*(*y*)*dz*)]

*s = ―――――――――――――――――――――*  (B2)

[±*erfc*(*y*)]*^1/4^*

This analytical result predicts the two types of solutions, decaying and periodical. The appearance of the latter stems from the fact that the expressions under the square root in (B2) can be either positive or negative. At large times, when *t→∞*, the function *erfc*(*y*)*→1*, and the integrals ∫*√erfc*(*y*)*dz and* ∫*√-erfc*(*y*)*dz* are either real or complex, respectively. Their sum in the numerator then gives either hyperbolic or trigonometric functions. A numerical evaluation of (B2) showed that the results appropriately approach the steady state solutions given by Eq. (12).

Interestingly, at *t→∞* the original PDE (9) in the main text transforms into a seminal Kolmogorov-Petrovskii-Piskunov-Fisher equation derived in 1937. Its most known solution is the travelling wave, which has never been applied to the consideration of calcium diffusion in the cytoplasm. Anco et al. (2011) recently applied a group foliation method and obtained several other explicit solutions for KPP-F equation. Remarkably, they show that the time-dependence is proportional to 1/*cos^2^* or 1/*cosh^2^*, which approach the steady state solutions given by (12). The existence of multiple solutions of a seminal KPP-F equation is not surprising. Of note, Polyanin & Zajtsev, (2012) enlist dozens of possible solutions even for a simple diffusion equation *C_t_* = *DC_rr_*. Some of them are expressed through hyperbolic of trigonometric functions.

Anco, S. C., Ali, S. and Wolf, T. (2011). Exact solutions of nonlinear partial differential equations by the method of group foliation reduction. SIGMA 7, 66-78.

Fisher, R. A. (1937). The wave of advance of advantageous genes, Ann. Eugenics 7, 353–369.

Holmes, M. H. (1995). Introduction to perturbation methods. Springer Verlag, Germany.

Polyanin, A. P., and Zaitsev, V. F. (2012). Handbook of Nonlinear Partial Differential Equations, Chapman & Hall/CRC Press, Boca Raton–London,

Kolmogorov, A. , Petrovskii, I., and Piskunov, N. (1937). A study of the diffusion equation with increase in the amount of substance, and its application to a biological problem. Bull. Moscow Univ., Math. Mech. 1, 1–25.

***C. The effects of ‘fast’ and ‘slow’ calcium buffers***

The concept of nanodomains originally evolved to explain the difference in the effects of calcium buffers – EGTA and BAPTA – upon calcium-activated K*^+^* channels. The two species were accordingly dubbed as slow and fast buffers, because they bind calcium with apparently 100-fold differences in the on-rate constants (Smith et al. 1984). Seemingly slow calcium binding by EGTA has been previously discussed (Mironova and Mironov, 2008). In summary, a doubly protonated EGTA (H_2_EGTA^2-^) is dominant at physiological pH but it cannot bind calcium efficiently, because *K_d_* *=* 4 M (Smith et al. 1984) indicates its extremely low affinity to calcium. HEGTA^3-^ has *K_d_* *=* 5 µM and can do this, but the concentration at normal pH is only 0.1 mM in 10 mM EGTA, 100 times smaller. At the ms-time scale a calcium ion is captured by the first buffer molecule it meets in the cytoplasm with the on-rate close to the diffusion limit. The rate of calcium binding is *k_on_*[Buffer]. An apparent 100-fold difference in *k_on_* values simply reflects the fact that [BAPTA]/[HEGTA^3-^] = 100 at equal nominal EGTA and BAPTA concentrations. EGTA can accommodate calcium with time by exchanging calcium for protons, but this proceeds very slowly. This explains why EGTA minimally disturbs fast calcium transients, but, at the same time, is effective in preventing calcium overload in e. g. whole-cell recordings. On the long time scale EGTA virtually eliminates possible deleterious long-lasting increases in cytoplasmic calcium. The same considerations are true for parvalbumin, where high-affinity Ca-binding sites at rest are occupied by Mg.

Smith PD, Liesegang GW, Berger RL, Czerlinski G, Podolsky RJ. 1984. A stopped-flow investigation of calcium ion binding by ethylene glycol bis(beta-aminoethyl ether)-N,N'-tetraacetic acid. Anal Biochem. 143: 188-195.

***D. Ca decay from the steady state.***

To assess the life-time of Ca gradients established around single channels, I set the steady state Ca given by Eq. (12) as initial condition and solved the time-dependent equations with the Crank-Nicolson method. Fig. S2A shows the results for 1D-diffusion and Fig. S2B present radial diffusion. Note that initial gradients are sharper and decay faster in the 3D-case; that can be explained by additional hyperbolic dependence (Ca values in Eq. (12) are divided by the distance to the channel).


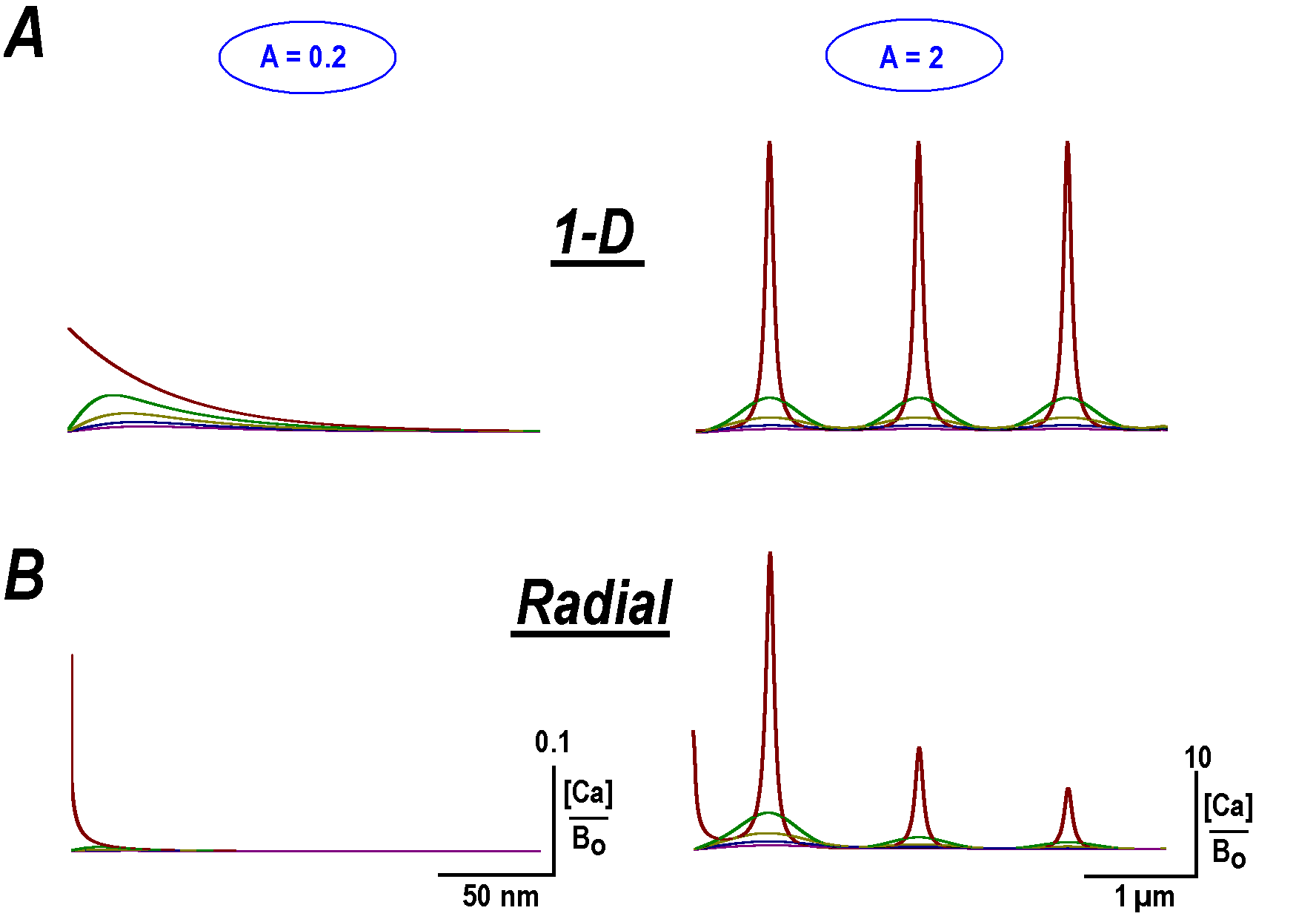


**Fig. S2. Dissipation of Ca gradients around single calcium channel after its closure**

Calcium profiles after setting ‘calcium fluxes’ to zero. Initial patterns (brown curves) correspond to the steady state solution for *A* = 0.2 and 2 as defined after Eq. (7). The values were chosen to bracket the critical value [Ca]*_o_*/*B_o_* = *A = 1* that separates decaying and periodic solutions. Time-decay is shown by differently colored curves calculated at 100 µs-time steps.

***E. The case of two buffers with different motilities.***

The introduction of the second Ca buffer gives an additional RD equation. I assume that Ca and the first buffer have equal motilities (*D_B1_/D_Ca_*) and the second buffer has a relative diffusion coefficient of *d* = *D_B2_/D_B1_*. The assumption simplifies the derivation but is not essential and does not modify the conclusions. Subtracting the equation for Ca in the steady state and the sum of the two buffer equations gives

(*c – f - dF*)*_xx_ = 0* (E1)

where *F* is the concentration of the second (Ca-free) buffer. Similar to Eqs. (7) and (8), in the steady state this gives

*0 = c_xx_ – c*[*c + 1 – A - dF*] (E2)

For *d* < 1 (the second buffer is much less mobile than the first one), we can treat the last term as perturbation. To zero order (E2) gives the two solutions (12) presented in the main text for single buffer. For the second buffer the steady state equation is

*dF_xx_ = -cF* (E3)

Here Ca unbinding is again neglected and *c* is the unperturbed Ca concentration that may have either a decaying or periodic pattern. The general solution of (D3) can be presented as

*F = F_1_* (*B_1_ + B_2_∫dx/F^2^_1_* ) (E4)

(Polyanin & Zajtsev, 2001), where a non-trivial particular solution of (E3) is *F_1_ = cosh* [(*z + w*)/*2*]/*sinh* [(*z + w*)/*2*] (see also Eq. (12)). The coefficients *B_i_* are determined from the boundary condition for the second buffer. I simply set *F_x=0_* = 0 i. e. the second buffer at the origin is fully saturated with Ca. Then Eq. (E3) presents a non-linear Schrödinger equation (see Supplement B), solvable in terms of special functions, which does not help much. I integrated (E2) numerically using the shooting method. The results for *d* = 0.1 and different values of *A* in Fig. S2 indicate that less mobile buffer modify both decaying and periodic Ca patterns such as they gain amplitude and become more extended. The result supports the trend indicated in the main text (a paragraph after Eq. (12)), stating that a decrease in buffer mobility increases the characteristic spatial scale. The calculations were done for *d* = 0.1 and can imitate the presence of mobile and immobile buffers. The considerations may cover many applications, because Ca-binding species in the cytoplasm can be pooled into the two buffer sorts - diffusible and fixed ones (Mironova & Mironov, 2008).


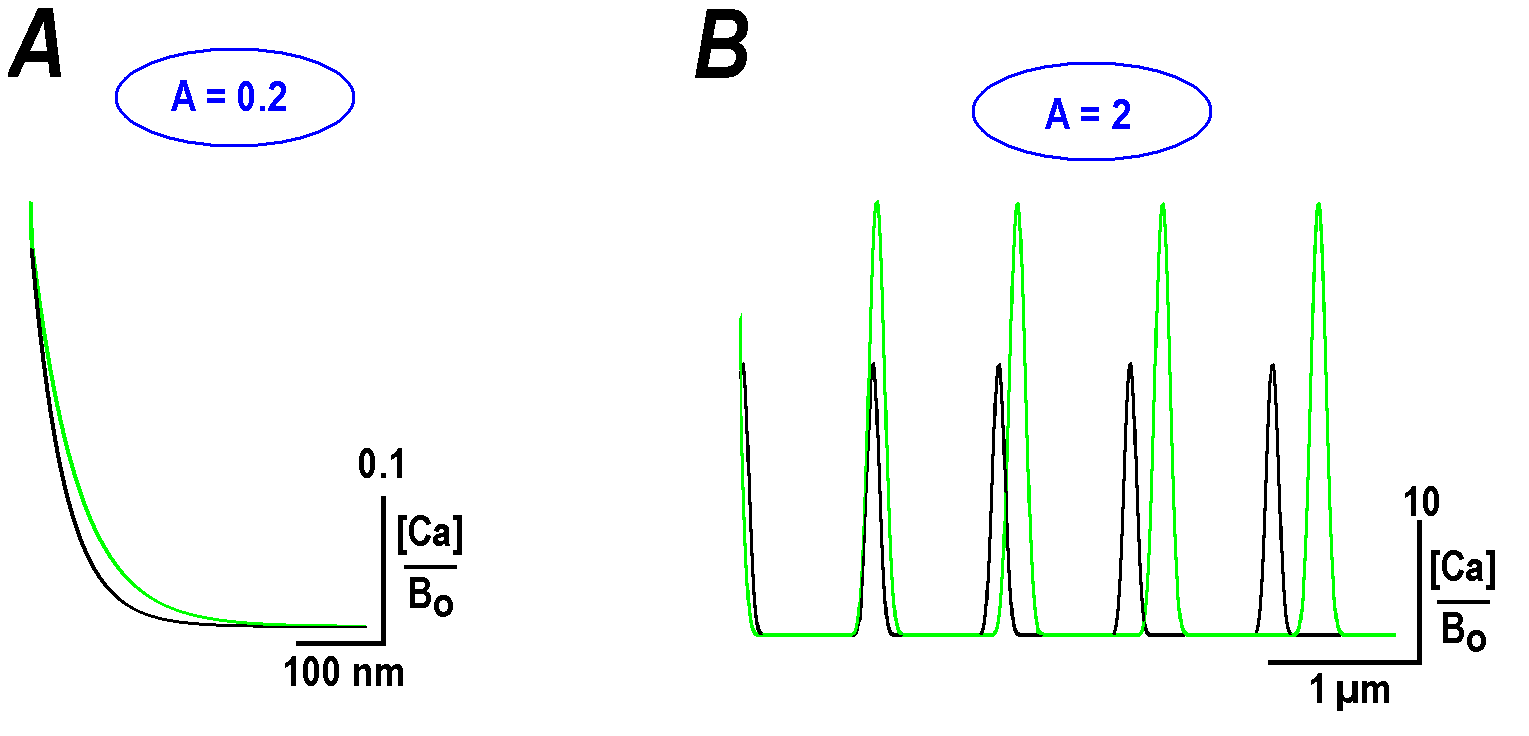


**Fig. S3. Calcium profiles in the presence of the two buffers with different mobilities.**

1D-calcium profiles in the steady state were calculated for ‘calcium fluxes’ corresponding to the parameter *A* = 0.2 (***A***) and 2 (***B***) as defined after Eq. (7) in the main text. The values were chosen to bracket the critical value [Ca]*_o_*/*B_o_* = *A = 1* that separates decaying and periodic solutions. Black curves represent calcium patterns for equal diffusion coefficients of Ca and mobile buffer. The green curves were obtained from Eq. (D2) after replacing the half of fast moving buffer with a less mobile buffer whose relative mobility was set to *d* = 0.1. Note that the calcium peaks become bigger and more extended. The periodic profiles had bigger spacing between calcium peaks.

Mironova, L. A., and Mironov, S. L. (2008). Approximate analytical time-dependent solutions to describe large-amplitude local calcium . Biophys. J. 94, 349-258.

Polyanin, A. P., and Zaitsev, V. F. (2001). Handbook of Ordinary Differential Equations, Chapman & Hall/CRC Press, Boca Raton–London.

***F. Reversible calcium binding***

To consider the effects of calcium unbinding in the steady state, I used Eq. (8) in the main text, written as

*c_t_ = c_xx_ – c*[*c + 1 – A erfc*(*x/2√t*)] *+ γ*(*1* – *f*) (F1)

where *γ = k_off/_k_on_B_o_ = K_d_/B_o_* is the rate constant for the Ca unbinding reaction. Using the normalized variables *s* = *c*/|*1 – A + γ*| and *z = x√*|*1* – *A + γ*| and the expression of Ca-free buffer *f* given by Eq. (7) in the main text, from Eq. (F1), I get two equations

*s_xx_* - *s*(*s + ß*) *+ γ_1_* = *0 A < 1,* with *γ_1_* = *γA/*(*1 – A + γ*)*^2^* (F2a)

*s_xx_* + *s* (*s - ß*) *- γ_2_* = *0, A > 1,* with  *γ_2_* = *γA/*(*A – 1 - γ*)*^2^* (F2b)

They differ from Eq. (10) only by the constant term. The fundamental solutions are the double-periodic Weierstrass functions (Abramowitz & Stegun, 1972; Atlas of Functions, 2009). This is not very useful, because no convenient approximation of such transcendent functions exists.

I sought for particular solutions using closely related Jacobi functions. They are specified by the index *m.* When *m* = *1*, the Jacobi functions are simply hyperbolic functions. Because a correlate of *1/sinh* is *ds*(*z│m*) (see Eq. 12)), I inserted a probe function

*s = b + a . ds*(*αz│m*)

into (F2). This specified the parameters *a* = (*2m – 1*)/(*5m^2^ - 5m + 2*), *b* = *A*(*2m – 1*)/*3,* and *α* = √*A/3*. For *m = 1,*  *a = 3α^2^, b =* (*α^2^*  - *1*)/*2* and *α^2^ = √1+4γ*, the explicit expressions for Ca concentration are

*γ 3/2* (*1 + γ*)*^2^*

*s = — + ———————— A < 1* (F3a)

*2 sinh^2^*[(*αz + w*)*/2*]

*γ 3/2* (*1 + γ*)*^2^*

*s = — + ——————— A > 1* (F3b)

*2 sin^2^*[±*αz + w*]

When Ca unbinding from buffer is slow, *γ* is close to *0* and *α* is close to *1,* and the expressions transform into the formulas (12)*.* When *γ* is finite, the steady state calcium has a small offset, increased amplitude and decreased width. As mentioned in the main text, in most cases *γ =* 0.005 << 1, and the effects of calcium unbinding can be skipped.

An Atlas of Functions. 2009. Oldham K, Myland J, Spanier, J. Hemisphere Publishing Company, 1987.

Abramovitz M., Stegun I.A. (eds.) 1972. Handbook of mathematical functions, 10 ed., NBS.

***G. The case of fast and slow Ca buffers***

Let us consider the effects of the two buffers with different Ca binding on-rates. In the steady state, the system of RD equation (Eq. (5)) extends to

*0 = c_xx_ – cf – µcφ*

*0 = f_xx_ – cf* (G1)

*0 = φ_xx_ – µcφ*

where *f* and *φ* are the normalized concentrations of the two Ca-free buffers. When the binding of Ca by the second buffer is slower by the factor *µ* *= k_on_,* *_φ_ < k_on, f_*, the equations attain the form typical for so called boundary layer problem with multiple (two) time-scales. It can be solved by different methods developed in the field (cf. Bender & Orszag, 1999).

In zero order *µ = 0* and the first two equations in (G1) give the Ca profiles described by Eq. (12) in the main text. In the first order by *µ*, the conservation condition (derived similarly to Eqs. (6) and (7) in the main text), is

*Φ = C – F– φ* (G2)

where *Φ* is the total concentration of the second (slower) buffer, *C* and *F* are the concentrations of free Ca and first (fast) buffer in the first order. The steady state equation for Ca reads then as

*C_xx_ =* (*cF+fC*) *–cφ =* [*C*(*2c - 1 - A*) + *c*(*Φ - φ + µφ*)] (G3)

where *A* is the initial calcium level at channel exit (Eq. (3)). Eq. (G3) and the third equation in (G1) for *φ* were solved for *µ* = 0.1 using the shooting method as in Supplements A and E*.* Fig. S4 shows the solutions obtained for *A* = 0.2 and 2. The results indicate that the inclusion of additional (slowly binding buffer) only slightly modify both the decaying and periodic solutions. Extension to the case of multiple binding species is straightforward.

The main result of this chapter is that the buffer with the fastest Ca binding gives the main contribution to the steady state Ca profiles. Therefore the buffer that moves and binds faster then others can be taken as initial approximation and the rest can be accounted for as described in this part and in Supplement E.

Bender C. M., Orszag S. A. (1999). Asymptotic Methods and Perturbation Theory. McGraw-Hill.


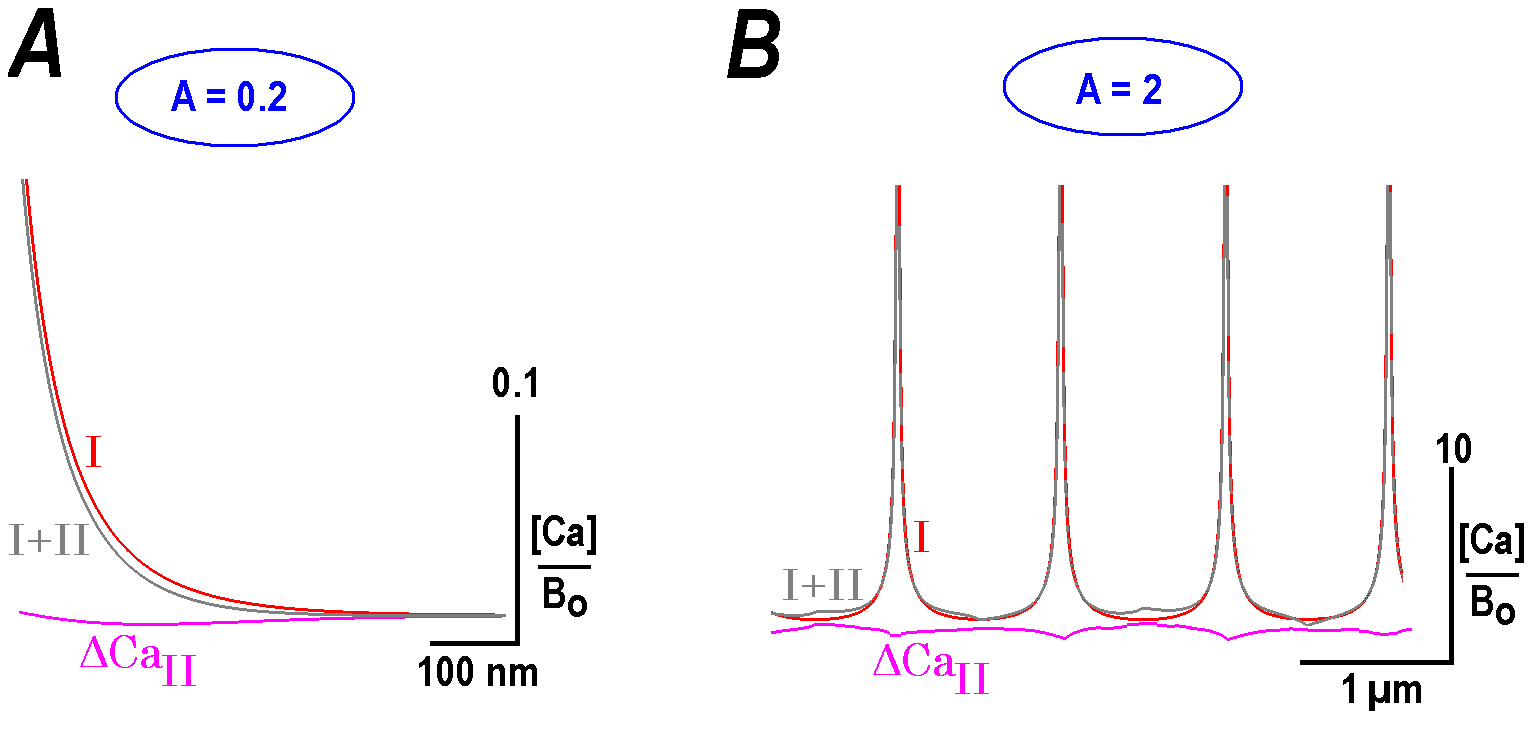


**Fig. S4. Calcium profiles in the presence of the two buffers with different on-rates.**

1D-calcium profiles in the steady state were calculated for ‘calcium fluxes’ corresponding to the parameter *A* = 0.2 (***A***) and 2 (***B***) as defined after Eq. (7) in the main text. The values were chosen to bracket the critical value *A = 1* that separates decaying and periodic solutions. Red curves (I) show the Ca patterns when only one buffer was present. The grey curves (II) were obtained from Eq. (G3), when the fast and slow buffers were present at equal concentrations (0.2 mM). The violet curves (ΔCa_II_) indicate decreases in free Ca caused by the presence of the slow buffer. The on-rate for slow buffer was taken 10-fold smaller than for the fast one.
